# Supplementary material for: Working nights and lower leisure-time physical activity associate with chronic pain in Southern African long-distance truck drivers: A cross-sectional study
Source: PLoS One. 2020 Dec 3;15(12):e0243366. doi: 10.1371/journal.pone.0243366 (PMC7714191; doi:10.1371/journal.pone.0243366)
Supplement: Attachment — Submitted filename: PONE-D-20-22799R1 r2r.docx [file pone.0243366.s001.docx]

**Reviewer #1:**

Prof Lemke, we were delighted to have you review our paper. Thank you for your

insightful comments and for your support in helping us strengthen our

manuscript.

**General Comments/Feedback:**

**There are opportunities for improving language/grammar in the**

**manuscript. For example, line 79 should read "associated with" rather than**

**"associating with".**

We have amended language and grammatical errors throughout the

manuscript.

**Introduction:**

**Overall, the section seems a bit thin...there isn't a strong theoretical or**

**practical (i.e., for prevention/intervention) basis/justification for exploring**

**the relationships that are queried in this study**

The introduction has been re-written to give a stronger theoretical

rationale for exploring chronic pain, and for exploring associations with

psychosocial factors, sleep and physical activity. The usefulness of these

data in informing health interventions for truck drivers has also been

discussed. Thank you for this observation. We feel the paper is much

stronger for the new introduction.

*“Trucking is a major industry and employer worldwide. In the US alone the*

*trucking industry generated almost $800 billion in revenue in 2018 and employed*

*3.5 million drivers [1]. In South Africa, due to the lack of waterways and the poor*

*rail services, road transport is essential to the national economy [2], and*

*transport workers represent a large group of employees: over 70,000 truck*

*drivers, who are mostly men [3].*

*As truck drivers contribute significantly to economies, both worldwide and in*

*South Africa, ensuring the health of this occupational group is important [4]. This*

*task requires effort, though, as truck drivers are at a greater risk of a multitude of*

*physical and mental health disorders compared to the general population [5–7].*

*Risk factors for non-communicable diseases such as cardiovascular disease,*

*diabetes and poor mental health easily result from long-distance driving due to*

*lack of physical activity, inability to eat healthily, and reduced or disrupted sleep*

*[8,9]. Obesity and reduced physical activity are also risk factors for chronic pain*

*[10,11], which is one of the most common causes of disability and reduced*

*quality of life [12,13]. Studies of truck drivers’ health to date have tended to focus*

*on cardiovascular health [14,15] yet chronic pain affects more Americans than*

*cardiovascular disease, diabetes and cancer combined [16].*

*The prevalence of pain in truck drivers ranges from 27-81% [8,17–20] and longdistance*

*truck drivers have a higher prevalence of pain than short-haul drivers*

*[19]. In addition to road accident-related injuries, prolonged sitting and exposure*

*to vibration, truck drivers are at high risk of non-traffic-related incidents too as*

*work tasks involve lifting and unloading of cargo [18–20]. The development of*

*chronic pain is more complicated than risk of injury, however, and is strongly*

*associated with psychosocial factors such as depression and previous trauma*

*[21]. Of the studies that have looked at psychosocial factors on drivers’ pain, the*

*factors have tended to be work-related e.g. job satisfaction, perceived work*

*stress, effort-reward balance [19,20]. Even when social support has been*

*included it has been in terms of social support from employers and colleagues*

*rather than from individuals’ personal lives [19]. In essence, studies investigating*

*pain in truck drivers have not explored psychosocial factors from an holistic point*

*of view.*

*In addition to assessing trait (rather than just work-related) psychosocial factors,*

*a comprehensive investigation of factors associating with pain in truck drivers*

*would also include sleep and physical activity. Truck drivers work night shifts,*

*likely reducing sleep and causing irregular sleep/wake times, and have low*

*physical activity due to long hours working [9]. Pain and sleep have a reciprocal*

*relationship [22,23]. Together, depressed mood, disrupted sleep and reduced*

*physical activity often associate with pain and reduced quality of life in general*

*populations [24,25]. The associations of these factors in a cohort of truck drivers*

*has not been examined, however.*

*Healthcare programmes specifically designed for truck drivers in sub-Saharan*

*Africa have been operating for the last 30 years [26]. These programmes have*

*typically focused on HIV testing and counselling and more recently have offered*

*primary health care services too [26]. Roadside Wellness Centres (RWC) and*

*mobile HIV clinics have been set up along driving routes in South Africa to*

*provide such services. An analysis of the up-take of RWC services*

*recommended that the services provided at the RWC needed to be designed for*

*the needs of truck drivers [27]. In order to design services for long-distance*

*drivers that promote health and quality of life, factors that associate with chronic*

*pain need to be determined. As such, the aim of this study was to assess*

*associations with a variety of factors including psychosocial, sleep, physical*

*activity and driving-related factors with the prevalence of chronic pain in truck*

*drivers. As long-distance drivers are more likely to work nights than shortdistance*

*drivers, and we wished to investigate the association between sleep*

*disruption and pain, we recruited long-distance drivers.”*

**There is a reference in your first paragraph for the "American Trucker**

**Association" - did the authors mean the American Trucking Associations?**

Thank you. Yes, we did mean the American Trucking Associations. We

have amended the reference.

**There are seemingly direct quotes that should have adequate in-text**

**citations to know which studies each came from.**

Thank you, we see your point. We have added references to several

sentences that previously only referred to the literature in the following

sentences.

**Materials and Methods:**

**More information regarding how participants were recruited, what day/time**

**drivers were recruited, the percent of drivers that were approached that**

**agreed to participate, etc. would be helpful.**

Extra detail about recruitment has been added to the methods (line 139).

*“Data were collected at North Star Alliance roadside wellness clinics (RWC) or*

*the nearby truck stops in Pomona and Soweto, which are in the Gauteng*

*province, and Bloemfontein, which is in the Free State province, of South Africa.*

*Recruitment took place from outside the RWC or at the truck stops on weekdays*

*during the clinic operating times, which varied from 4pm (Bloemfontein) to 8pm*

*(Pomona). Recruitment in Soweto took place during weekdays and also on*

*weekends until 3pm. There were several methods used to recruit truck drivers:*

*invitation cards were handed out at the truck stops to individuals and groups,*

*cards were placed on windscreens and at recruiting companies. Sometimes a*

*group would form when a recruiter was speaking to one or two drivers. As such,*

*it was not possible to record accurately the number of drivers agreeing to*

*participate and those declining.”*

Please note that our inability to record the percentage of those agreeing to

participate has been acknowledged as a limitation (line 448).

**It isn't clear whether participants were offered incentives.**

We have added this information to line 156.

*“Participants were given 150 ZAR and a T-shirt for taking part.”*

**If drivers did not fast, it may have possibly impacted their c-reactive protein**

**levels (leading to an important confound). I am not certain whether this is**

**the case, but this should be looked into by the authors and, if this indeed a**

**possible confound, it should be acknowledged as a limitation.**

It is correct that the drivers did not fast before having their C-reactive

protein levels measured. Fasting is not required before assessment of Creactive

protein (please see laboratory instructions below). Thus, with

respect, we do not believe this is a confounding factor.

*https://medlineplus.gov/lab-tests/c-reactive-protein-crp-test/*

*https://www.mayoclinic.org/tests-procedures/c-reactive-proteintest/*

*about/pac-*

*20385228#:~:text=There%20are%20no%20preparations%20for,can%20af*

*fect%20your%20CRP%20level.*

**In the United States, there are many different types of truck driver routes**

**(e.g., long-haul), and the type of route can have important implications for**

**health and safety. Is this also the case in South Africa? If so, the types of**

**drivers that participated and how these characteristics may have impacted**

**the findings should be discussed in the manuscript.**

Thank you for highlighting the lack of clarity around the drivers in the

study. We recruited long-distance truck drivers and we have amended the

title to reflect this. This detail had been omitted from the inclusion criteria,

which has now been rectified (line 154).

We have addressed the relevance of the type of drivers in the introduction

and discussion including pointing out that long-distance drivers are at

greater risk of health problems than short-haul drivers.

In the introduction (line 84):

*“Risk factors for non-communicable diseases such as cardiovascular disease,*

*diabetes and poor mental health include factors that easily result from longdistance*

*driving including lack of physical activity, poor quality food, and reduced*

*or disrupted sleep [8,9].”*

And on line 94:

*“The prevalence of pain in truck drivers ranges from 27-81% [8,17–20] and longdistance*

*truck drivers have a higher prevalence of pain than short-haul drivers*

*[19].”*

And after the aim on line 131:

*“As long-distance drivers are more likely to work nights than short-distance*

*drivers, and we wished to investigate the association between sleep disruption*

*and pain, we recruited long-distance drivers.”*

And in the discussion Line 383:

*“Still, long-distance truck drivers are known to be more at-risk for chronic pain*

*than short distance truckers (19). For instance, a recent study performed among*

*123 truck drivers working for the same company in Canada reported that*

*prevalence of musculoskeletal pain was twice as high among long-distance*

*drivers compared to short-distance truckers (19). Interestingly, the authors found*

*that musculoskeletal pain among long-distance drivers was related to lifestyle*

*(physical inactivity, smoking, reduced sleep) and psychosocial factors, while*

*musculoskeletal pain among short-distance drivers was mainly related to*

*physical risk factors (lifting and vibration).”*

**Discussion:**

**It would be helpful to readers outside of the South African context to**

**provide further insight into the trucking industry in South Africa, and**

**especially the key similarities and differences with other trucking**

**industries globally.**

On line 394, we preceded discussion of stress and depression in longdistance

drivers, with these similarities:

*“Worldwide, including in South Africa, long-distance truck drivers are subject to*

*challenging working conditions including long working hours, pressure to meet*

*deadlines, insufficient rest, and exposure to violence such as high-jacking of*

*cargo (8,28,52–54).”*

And following discussion of health care programmes for truck drivers on

line 481, this difference was added:

*“The one difficulty in South Africa, however, is that most long-distance drivers are*

*non-South Africans (whereas in other countries drivers are largely citizens),*

*which has implications for retaining drivers in care. This issue can be overcome*

*as illustrated by a Trucking Association having an electronic health passport*

*system (78).”*

**The authors should reflect on the findings of their work in reference to**

**truck driver health and wellness prevention/intervention programs - what**

**do these findings contribute to actually improving the quality of life of**

**professional drivers?**

On line 402, we added about added a suggestion for mental health assessment:

*“In the present study, the prevalence of depressive symptoms was higher in the*

*truck drivers than males in the general South African population (40% vs 24%)*

*(57). This finding suggests that assessment of mental health in truck drivers, and*

*potential referral, could be a useful addition to roadside health services.”*

We made the following suggestions for expanding services at roadside wellness

clinics, line 472:

*“The present study has provided data for the first time on the relationships*

*between chronic pain, sleep, depression and physical activity in long-distance*

*truck drivers. These data support interventions to improve physical and*

*psychosocial health of long-distance truck drivers who are an important part of*

*both the local economies in Southern Africa but also globally. The drivers were*

*recruited at truck stops where a health programme is already running. Health*

*programmes dedicated to truck drivers exist in Sub-Saharan Africa too but most*

*are dedicated to HIV prevention and diagnosis (26). Wellness centres and*

*exercise rooms have been proposed at trucker stops (77) and these data support*

*such interventions. Health programmes could be expanded to assess mental*

*health with drivers referred as necessary. The one difficulty in South Africa,*

*however, is that most long-distance drivers are non-South Africans (whereas in*

*other countries drivers are largely citizens), which has implications for retaining*

*drivers in care. This issue can be overcome as illustrated by a Trucking*

*Association having an electronic health passport system (78).”*

*And in the conclusion, line 512:*

*“*Potential improvements to current health programmes for truck drivers include

health screening that assesses pain, mental health, sleep and activity. The

feasibility of exercise facilities at, or nearby, truck stops should also be explored.”

_______________________________________________________________________

**Reviewer #2**

Thank you for your positive response to our manuscript and for your ideas on

how to improve it.

**Introduction:**

**The authors need to provide much more background literature in relation to**

**the working conditions of truck drivers.**

We have added commentary regarding the working conditions of truck

drivers to the introduction, and in line with Reviewer 1’s suggestion, we

have added commentary to the discussion too.

Line 83

*“…truck drivers are at a greater risk of a multitude of physical and mental health*

*disorders compared to the general population [5–7]. Risk factors for noncommunicable*

*diseases such as cardiovascular disease, diabetes and poor*

*mental health easily result from long-distance driving due to lack of physical*

*activity, inability to eat healthily, and reduced or disrupted sleep [8,9].”*

Line 96

*“In addition to road accident-related injuries, prolonged sitting and exposure to*

*vibration, truck drivers are at high risk of non-traffic-related incidents too as work*

*tasks involve lifting and unloading of cargo [18–20].”*

Line 110

*“Truck drivers work night shifts, likely reducing sleep and causing irregular*

*sleep/wake times, and have low physical activity due to long hours working [9].”*

Line 394

*“Worldwide, including in South Africa, long-distance truck drivers are subject to*

*challenging working conditions including long working hours, pressure to meet*

*deadlines, insufficient rest, and exposure to violence such as high jacking of*

*cargo (8,28,52–54)”.*

**The reviewer suggests that the authors do not include their recent study in**

**the introduction - move it and refer to it in the discussion section.**

The reference to the previous study has been moved out of the

introduction and into the discussion.

**The authors should not include the last paragraph of the introduction - it**

**should be moved to the materials and methods section.**

We have moved the last paragraph of the introduction into the materials

and methods section. We have replaced the paragraph with two

sentences to articulate the aims of the study.

Line 126

*“As such, the aim of this study was to assess associations with a variety of*

*factors including psychosocial, sleep, physical activity and driving-related factors*

*with the prevalence of chronic pain in truck drivers. As long-distance drivers are*

*more likely to work nights than short-distance drivers, and we wished to*

*investigate the association between sleep disruption and pain, we recruited longdistance*

*drivers.”*

**Overall, the authors need to provide much more of a rationale for the**

**importance of the study - much more literature support needs to be**

**provided.**

The introduction has been re-written to give a stronger theoretical

rationale for exploring chronic pain, and for exploring associations with

psychosocial factors, sleep and physical activity. Thank you for this

observation. We feel the paper is much stronger for the new introduction.

*“Trucking is a major industry and employer worldwide. In the US alone the*

*trucking industry generated almost $800 billion in revenue in 2018 and employed*

*3.5 million drivers [1]. In South Africa, due to the lack of waterways and the poor*

*rail services, road transport is essential to the national economy [2], and*

*transport workers represent a large group of employees: over 70,000 truck*

*drivers, who are mostly men [3].*

*As truck drivers contribute significantly to economies, both worldwide and in*

*South Africa, ensuring the health of this occupational group is important [4]. This*

*task requires effort, though, as truck drivers are at a greater risk of a multitude of*

*physical and mental health disorders compared to the general population [5–7].*

*Risk factors for non-communicable diseases such as cardiovascular disease,*

*diabetes and poor mental health easily result from long-distance driving due to*

*lack of physical activity, inability to eat healthily, and reduced or disrupted sleep*

*[8,9]. Obesity and reduced physical activity are also risk factors for chronic pain*

*[10,11], which is one of the most common causes of disability and reduced*

*quality of life [12,13]. Studies of truck drivers’ health to date have tended to focus*

*on cardiovascular health [14,15] yet chronic pain affects more Americans than*

*cardiovascular disease, diabetes and cancer combined [16].*

*The prevalence of pain in truck drivers ranges from 27-81% [8,17–20] and longdistance*

*truck drivers have a higher prevalence of pain than short-haul drivers*

*[19]. In addition to road accident-related injuries, prolonged sitting and exposure*

*to vibration, truck drivers are at high risk of non-traffic-related incidents too as*

*work tasks involve lifting and unloading of cargo [18–20]. The development of*

*chronic pain is more complicated than risk of injury, however, and is strongly*

*associated with psychosocial factors such as depression and previous trauma*

*[21]. Of the studies that have looked at psychosocial factors on drivers’ pain, the*

*factors have tended to be work-related e.g. job satisfaction, perceived work*

*stress, effort-reward balance [19,20]. Even when social support has been*

*included it has been in terms of social support from employers and colleagues*

*rather than from individuals’ personal lives [19]. In essence, studies investigating*

*pain in truck drivers have not explored psychosocial factors from an holistic point*

*of view.*

*In addition to assessing trait (rather than just work-related) psychosocial factors,*

*a comprehensive investigation of factors associating with pain in truck drivers*

*would also include sleep and physical activity. Truck drivers work night shifts,*

*likely reducing sleep and causing irregular sleep/wake times, and have low*

*physical activity due to long hours working [9]. Pain and sleep have a reciprocal*

*relationship [22,23]. Together, depressed mood, disrupted sleep and reduced*

*physical activity often associate with pain and reduced quality of life in general*

*populations [24,25]. The associations of these factors in a cohort of truck drivers*

*has not been examined, however.*

*Healthcare programmes specifically designed for truck drivers in sub-Saharan*

*Africa have been operating for the last 30 years [26]. These programmes have*

*typically focused on HIV testing and counselling and more recently have offered*

*primary health care services too [26]. Roadside Wellness Centres (RWC) and*

*mobile HIV clinics have been set up along driving routes in South Africa to*

*provide such services. An analysis of the up-take of RWC services*

*recommended that the services provided at the RWC needed to be designed for*

*the needs of truck drivers [27]. In order to design services for long-distance*

*drivers that promote health and quality of life, factors that associate with chronic*

*pain need to be determined. As such, the aim of this study was to assess*

*associations with a variety of factors including psychosocial, sleep, physical*

*activity and driving-related factors with the prevalence of chronic pain in truck*

*drivers. As long-distance drivers are more likely to work nights than shortdistance*

*drivers, and we wished to investigate the association between sleep*

*disruption and pain, we recruited long-distance drivers.”*

**Materials and methods:**

**The authors need to provide more details about the data collection**

**process. How were the truck stops chosen? Why?**

More detail has been added about the data collection process including

why the truck stops were chosen (line 139 onwards).

*“Data were collected at North Star Alliance (NSA) wellness clinics or the nearby*

*truck stops in Pomona in the Gauteng province, and Bloemfontein, which is in the*

*Free State province, of South Africa.These stops were chosen because of the*

*close proximity of the wellness clinic for referral and the and the high volume of*

*truck drivers in the vicinity. Recruitment also took place in Soweto, Gauteng*

*Province which was a busy truck stop but not located near the NSA wellness*

*clinic, in order to include more South Africans in the survey. Recruitment took*

*place from outside the clinic or at the truck stops on weekdays during clinic*

*operating times, which varied from 4pm (Bloemfontein) to 8pm (Pomona).*

*Recruitment in Soweto took place during weekdays and also on weekends until*

*3pm. There were several methods used to recruit truck drivers: invitation cards*

*were handed out at the truck stops to individuals and groups, cards were placed*

*on windscreens and at recruiting companies. Sometimes a group would form*

*when a recruiter was speaking to one or two drivers. As such, it was not possible to record accurately the number of drivers agreeing to participate and those declining.”*

**More details need to be provided in the "Chronic pain" section. Was a valid**

**and reliable scale used, etc.?**

The binary data of having chronic pain or not were gained from one

question using a standard time frame for determining chronic pain (Treede

et al., 2019) and as such, no scale was used. The following text has been

added regarding determining chronic pain.

Line 170

*“We used a simple but standard method for determining chronic pain:*

*chronic pain was classified as having had pain at any location for at least*

*the last three months (Treede et al., 2019). Participants were only asked*

*one question. They were asked to indicate any locations of pain from a list*

*of body parts, where they had had pain or discomfort for more than three*

*months. The data on location of pain sites has been presented elsewhere*

*(Lalla-Edward et al., 2019). Here the binary data of having chronic pain or*

*not were used.”*

**More rationale needs to be provided for the "exploratory analyses", both in**

**the introduction and analyses section. Why was it included in the present**

**study?**

Thank you for highlighting the need for extra clarity here. This text has

been added to the introduction (line 108):

“*In addition to assessing trait (rather than just work-related) psychosocial factors,*

*a comprehensive investigation of factors associating with pain in truck drivers*

*would also include sleep and physical activity. Truck drivers work night shifts,*

*likely reducing sleep and causing irregular sleep/wake times, and have low*

*physical activity due to long hours working [9]. Pain and sleep have a reciprocal*

*relationship with impaired sleep predicting pain, and pain impairing sleep [22,23].*

*Together, depressed mood, disrupted sleep and reduced physical activity*

*associate with pain and reduced quality of life [24,25]*.”

And this text has been added to the data analysis section (line 260)

(New text in italics)

“In addition, as there is a known relationship between sleep, pain and depression

(33), we ran exploratory analyses including sleep quality and depression levels

with the significant main effects found in our model *(working nights and leisuretime*

*physical activity). That is, to investigate the hypothesis of a relationship*

*between pain, sleep, depression and activity, we investigated whether 1) sleep*

*quality, sleepiness and depression levels were actually worse in those working*

*two or more nights a week compared to those working one or fewer nights a*

*week and 2) whether depression and sleep were worse in those with lower*

*leisure-time physical activity*.”

**Discussion:**

**Authors use "We" too much - refer to it as "The present study" or**

**something similar.**

We have replaced multiple instances of the use of “We” in the discussion.

**The authors do a good job of relating their findings to previous studies;**

**however, the authors need to do a better job and expand more on the realworld**

**implications of the findings. The reviewer recommends that the**

**authors provide much more in terms of the implications of the findings.**

**What are potential viable solutions for addressing the issues among truck**

**drivers in South Africa?**

Thank you, this is a really good point. In addition, to the conclusion

described below, the following paragraph has been added to the

discussion regarding the real-world implications of the findings.

Line 472

*“The present study has provided data for the first time on the relationships*

*between chronic pain, sleep, depression and physical activity in long-distance*

*truck drivers. These data support interventions to improve physical and*

*psychosocial health of long-distance truck drivers who are an important part of*

*both the local and global economies. The drivers were recruited at truck stops*

*where a health programme is already running. Health programmes dedicated to*

*truck drivers exist in Sub-Saharan Africa too but most are dedicated to HIV*

*prevention and diagnosis (26). Wellness centres and exercise rooms have been*

*proposed at trucker stops (77) and these data support such interventions. Health*

*programmes could be expanded to assess mental health with drivers referred as*

*necessary. The one difficulty in South Africa, however, is that most long-distance*

*drivers are non-South Africans (whereas in other countries drivers are largely*

*citizens), which has implications for retaining drivers in care – especially where*

*chronic care is required. This issue can be overcome as illustrated by a Trucking*

*Association having an electronic health passport system (78) and a strong*

*referral network for continuous access across major routes.”*

**The reviewer recommends a "Conclusions" section that gives a final "big**

**picture" implications of the study and findings.**

Thank you for highlighting that the big picture needed emphasising. We

have added a conclusions section with implications of the findings

including recommendations for health services and the law.

Line 505

*“Chronic pain is more prevalent in the United States than diabetes,*

*cardiovascular disease and cancer combined (16), and results in disability and*

*reduced quality of life (12,13). In this first study of factors associated with chronic*

*pain in male long-distance truck drivers, working two or more nights a week and*

*lower leisure-time physical activity were associated with having chronic pain. The*

*findings here support the inter-relatedness of pain, sleep, mood and physical*

*activity as working nights also associated with worse sleep and greater*

*depressive symptoms, and lower leisure-time activity associated with worse*

*sleep quality. Potential improvements to current health programmes for truck*

*drivers include health screening that assesses pain, mental health, sleep and*

*activity. The feasibility of exercise facilities at, or nearby, truck stops should also*

*be explored. Since both sleep deprivation and pain could result in a multitude of*

*negative consequences (including road accidents) stronger laws are*

*recommended that govern working hours, number of hours on the road (possibly*

*monitored through trackers in vehicles) and requirements on truck owners to*

*ensure that their drivers have access to proper sleeping (and possibly food and*

*exercise) facilities along their trucking routes. Truck driving contributes*

*significantly the global economy and promoting health in truck drivers in these*

*ways is not only logical strategically but has the potential to reduce disability and*

*improve quality of life in this at-risk occupational group (4).”*
